# Deep neural network-based analysis of the impact of ambidextrous innovation and social networks on firm performance

**DOI:** 10.1038/s41598-023-36920-9

**Published:** 2023-06-26

**Authors:** Xinyuan Zhang, Chee Heong Quah, Mohammad Nazri Bin Mohd Nor

**Affiliations:** Department of Management, Faculty of Business and Economics, University of Malaya, 50603 Kuala Lumpur, Malaysia

**Keywords:** Environmental social sciences, Space physics

## Abstract

The motivation for analyzing the impact of deep neural networks on enterprise performance is mainly due to the continuous deepening of enterprise information construction, shifting from traditional paper-based data acquisition methods to electronic data management. The data generated by the sales, production, logistics and other links of enterprises is also becoming increasingly large. How to scientifically and effectively process these massive amounts of data and extract valuable information has become an important issue that enterprises need to solve. The continuous and stable growth of China's economy has promoted the development and growth of enterprises, however, it has also made enterprises face a more complex competitive environment. The question of how to improve the performance of enterprises to enhance their competitiveness in the market has become a major issue to be addressed in the face of fierce competition and to ensure the long-term development of enterprises. In this paper, based on the research of firm performance evaluation, deep neural network is introduced to analyse the influence of ambidextrous innovation and social network on firm performance, and the theories of social network, ambidextrous innovation and deep neural network are sorted out and analysed, and a deep neural network-based firm performance evaluation model is established, and finally the sample data is obtained using crawler technology, and then the response values are analysed. Innovation and the improvement of the mean value of social networks are helpful to firm performance.

## Introduction

At present, enterprises all over the world pay great attention to the cultivation of talents, while China is now the key period of enterprise restructuring, the cultivation of talents is the most important. After several years of efforts, the construction of the company's workforce has begun to bear fruit, and has played a positive role in promoting the development of the company. However, there are still some problems with our current personnel management system, and in practice, there are also some very difficult to manage and difficult to manage problems. The reason for this is mainly due to the lack of meticulous management of personnel in enterprises, the work proposed is mostly rules and regulations, the lack of operable process management and supervision mechanisms, mostly subjective, relying on past management experience, resulting in imperfect management^1^. With the continuous improvement of the enterprise management system today, the evaluation of employees' daily work performance is receiving increasing attention and is used as an important reference for staff retention, party membership and examination^2^. However, at present, there are more problems in the evaluation of employees in China's enterprises. The reason for this is that the performance evaluation system of the company's employees is not sound^3^. The company's staff system has undergone several major changes and has now formed an overall system with a number of important elements such as compilation, selection, probation, appraisal, rewards and punishments as its core, which has played a good role in promoting the development of the staff team. However, so far, these systems have only initially outlined a general framework and pointed out a direction, while in the process of implementation and enforcement, there are still problems of poor operability and The appraisal rules are not perfect and comprehensive^4^. At present, the performance evaluation indicators for our employees are not sound. The company's staff management system does not have a detailed performance evaluation system specifically for different employees, and each department has to determine its own actual situation, but many companies have a problem with their performance evaluation indicators, that is, the correlation between the performance indicators and the employees' work objectives is not high. Secondly, it is difficult to quantify the indicators; thirdly, there is a lack of scientific analysis, and most of the indicators are not clearly defined in the evaluation indicators, making it difficult to reflect the actual performance of employees in different positions and posts; fourthly, there is no unified standard, leading to a lack of authority in the evaluation results^5^.

To solve the above problems, it is necessary to construct a standard, scientific and objective evaluation model^6^. This paper further delves into the mechanism of social networks' influence in technology innovation and entrepreneurship. In recent years, many scholars have recognised the role of social networks in technological innovation entrepreneurship, and their research is becoming increasingly sophisticated. Social networks support entrepreneurs throughout the entire process, from the incubation of technological innovation, through the growth process, to future development. An overview of the current market situation shows that there are many entrepreneurs with significant resources, but the business performance of their companies has been poor, the reason for this phenomenon is a common problem in academia in recent years^7^. Entrepreneurs can access the resources they need to survive and thrive from their social networks^8^. Now, it is more about academics.

For the analysis of the impact of dual innovation based on deep neural networks and social networks on corporate performance, we can conclude that dual innovation based on deep neural networks can promote technological and product innovation in enterprises, and has a significant positive impact on corporate performance. Social networks can help enterprises enhance their information acquisition, resource sharing, and collaborative innovation capabilities, improving their competitiveness and performance.

## Introduction to theories

### Social network theory

The novelty of analyzing the impact of dual innovation and social networks on corporate performance based on deep neural networks is a research issue that involves innovation, social networks, and corporate performance. Deep neural networks can be applied to many different problems and have high fitting and prediction abilities. In this case, it may be used to mine internal and external data of the enterprise to predict its future performance, or to better understand its current performance. Specifically, deep neural networks can learn the characteristics of enterprises based on historical data, such as products, market environment, marketing strategies, etc., in order to predict future sales, customer satisfaction, and other indicators. This helps enterprises make more precise decisions, improve efficiency and effectiveness. In the mid-1970s, the concept of social networks was gradually developed. Mitchell (1974) saw social networks as a system of social relations consisting of interactive links between individuals or groups^11^, and in 1908 the sociologist Zimmer first introduced the concept of "networks". The emergence of the "weak relationship" theory is an important feature that has led to the widespread use of social networks in various fields. In this paper, we analyse the research on social networks at home and abroad, and believe that the most representative social network research can be divided into three major schools of thought: the strong and weak ties school, the structural holes school and the social capital school^12^.

1. Strong and weak ties

Granovetter founded the theory of weak ties and introduced the concept of "embeddedness". In this paper, Granovetter classifies social networks into strong and weak ties, which are considered to be weak in their role of information transfer. Strong and weak ties are different in different contexts, whereas strong ties focus on relationships within groups and within organisations. Weak relationships focus on building connections between the objects being connected^13^.

(2) Constructing networks

Ronald Bert Bert and Ronald Bert (1992) suggest that 'structural holes' refer to the absence of connections in a network, i.e. poorly connected, resulting in a 'flat hole' in the network^14^.

Network nodes can use the holes in the network to obtain the corresponding information, so as to develop and grow themselves, and become the centre of the network, network heterogeneity, network density and network core are important indicators to measure the density of the network, the greater the network density, the higher the density of the core network, the greater the density of the network^15^.

(3) Social resources

With the "explosion" of Internet information, web crawler is gradually known to people and has been applied to many fields of social life. As a technology of automatically collecting web page data, many people do not know what scenarios web crawler can be applied to. In fact, most application scenarios that rely on data support are inseparable from web crawler, including search engines, public opinion analysis and monitoring, aggregation platforms, travel software, etc. The search engine is one of the most important application scenarios of the general web crawler. It will take the web crawler as the most basic part—the Internet information collector, and let the web crawler automatically grab data from the Internet. For example, Google, Baidu, Bing and other search engines use web crawler technology to collect massive data from the Internet. On the basis of Granovetter's "weak relationship strength assumption", the famous sociologist Lin Nan explored this in depth, and thus formed the social resource theory. In this theory, it is argued that the resources of a network are not owned by individuals, but by the wealth, power and fame of the social network. Based on this, Linnan scholars have put forward three hypotheses about social resources, which have been tested. (1) social status is significantly and positively related to access to social resources; the higher the social status of an individual or group, the more resources are acquired; (2) the strength of weak association hypothesis; the heterogeneity of social networks is significantly and positively related to the amount of access to resources. Poorer connections can be used to obtain more social resources; (3) the influence of social resources is assumed; the effect of instrumental behaviour is significantly related to social resources, i.e.: the more social resources the user has, the more desirable the instrumental behaviour is ^15^.

### Ambidextrous innovation theory

Exploratory innovation is a new kind of innovation that is characterised by search, variation, creation, experimentation and risk-taking, and is a quest to adapt to new market needs. Exploratory innovation helps to identify new market needs and seek new opportunities for growth, which can lead to radical changes in an entire industry^16^.

Companies can create more value by carrying out exploratory innovation activities to fill existing market gaps and guide customers' needs on the supply side. As domestic and international markets continue to develop and technology advances at a rapid pace^18^, companies are faced with the needs of rapidly changing industrial technologies and consumer markets, while undertaking exploratory innovation activities facilitates their development. In the new market conditions, companies are able to maintain their core competencies and by carrying out innovative activities, they can greatly improve their core competencies, seize market heights, control the dominant products in the industry, improve the quality of their products, increase their voice and thus gain economic benefits. The company undertakes pioneering innovations to develop new products in the market. New services refer to the provision of goods or services to customers on the supply side and their orientation to achieve control over them^17^.

However, successful innovation can also significantly increase a company's market share and cash flow, which can contribute significantly to the company's performance. Imitation is often used, especially in the early stages of a company's development. The short-term benefits of new acquisitions and, at a later stage, disruptive innovation of new products and services can lead to greater gains for the company, the acquisition of future core competencies and a corresponding voice and industrial advantage^18^.

However, innovation is a high-investment, high-risk and high-risk innovation activity, and therefore, when carrying out innovation, it is necessary to reintegrate and reallocate its resources, which leads to a high level of risk. Moreover, when carrying out innovation activities, its investment is also higher, which requires companies to invest more money. However, if firms become overly obsessed with the high returns of exploratory innovation and neglect the current high investment, they will fall into the 'exploratory' dilemma. From the perspective of evolutionary theory, van der Ven and Ball (1995) point out the impact on firm performance due to the large amount of new knowledge that differs from the original body of the firm. The firm itself has a very limited absorptive capacity and is unlikely to use the new knowledge to its full potential, making it difficult for it to circulate in the firm. Yet the positive impact of innovation on firm performance is significant. This paper argues that during China's economic transition, a larger market environment, a more inclusive innovation capacity, a greater The exploratory innovations undertaken by firms are all rewarded accordingly, and exploratory innovations play a pivotal role in the improvement of firm performance. The primary objective of innovation is to break away from the current market competition, enter a new field, open up new markets and offer new products and services to customers in order to achieve a leading competitive advantage, thereby significantly increasing the company's sales performance, sales revenue, market share and, finally, company performance.

## Research methodology design

### Theoretical model construction

Based on the above analysis of the theoretical review of the relationship between social networks, ambidextrous innovation and corporate performance, it can be concluded that social networks and ambidextrous innovation have a positive impact on job performance, and data analysis plays a mediating role in this regard.

**Figure 1:**
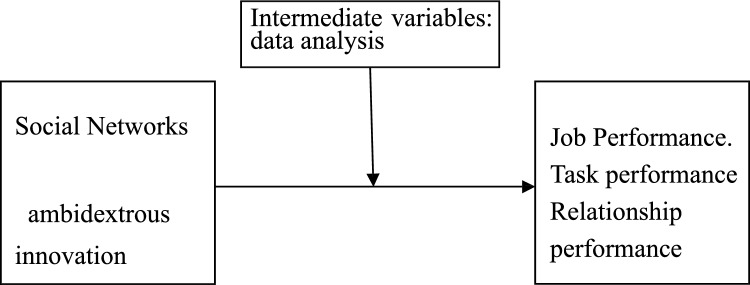
Theoretical model of the impact of social networks on firm performance.

Social networks can be combined with deep neural networks through the following methods: data sources: The online activities and behaviors of thousands of users on social networks generate a large amount of data, which can be used as training data sources for deep neural networks, including text, images, and multimedia content. User modeling: Deep neural networks can be used to model and predict users. User data in social networks can provide a large amount of information, such as user interests, social networks, user attributes, etc. These data can be applied to various deep learning models to predict user behavior and interests. In view of the above analysis, this paper will further explore the relationship between the three factors through an empirical approach and explore the impact of social networks and ambidextrous innovation on firm performance. The theoretical model developed in this paper is shown in Fig. 1.

### Construction of a deep neural network model

(1) Determining the network layers

Theoretical analysis shows that any closed interval can be approximated by a deep neural network containing one layer, so that a three-layer deep neural network can be mapped arbitrarily between N dimensions. It has the advantages of good structure, simplicity, ease of operation and short running time, and is now a widely used structural model for neural networks. Corporate performance is the performance of a company in achieving its goals and mission. With the development of the Internet and technological progress, more and more enterprises are beginning to apply technologies such as digitization and data analysis to enterprise management to improve enterprise performance. As a powerful artificial intelligence technology, deep neural networks have the advantage of processing large amounts of data and mining the potential value of data, so they are widely used by enterprises in various fields.

Deep neural networks can be used to improve the performance and efficiency of social network platforms, including content recommendation, user relationship modeling, and advertising placement. For example, deep neural networks can be used to improve the accuracy of content recommendations and measure the effectiveness of different advertising strategies. In fact, when the number of hidden layers and neurons is infinite, the actual output of the network can be infinitely close to the corresponding expected output, but in this case the overall generalisation performance will be reduced, resulting in "overfitting" and no practical value. Therefore, it is important to reduce the number of hidden layers and neurons within the accuracy range in order to increase the structural density of the neural network.

In some cases, exploratory innovation can bring many benefits to enterprises, such as generating new products or services, expanding new markets, and improving production efficiency. When these achievements bring higher returns, the enterprise may give corresponding rewards. However, in some cases, enterprises may face certain risks and challenges in exploratory innovation, such as the need for more investment and immature new technologies. In this situation, even if exploratory innovation fails, the enterprise should still receive recognition and support, as the experience and knowledge learned through innovation may have a positive impact in the future. In short, exploratory innovation by enterprises is often considered one of the important means to promote their development, and the rewards brought by innovation are usually proportional to the risks. Therefore, enterprises should weigh the pros and cons based on their own situation and develop appropriate innovation strategies. To this end, this thesis proposes a three-layer deep neural network, namely an input layer, a hidden layer and an output layer.

(2) Determining the number of input and output nodes of the network

Combined with the established comprehensive firm performance index assessment system, the input and output of the network were determined in detail.

In the deep neural network, the number of nodes in the input layer is the same as in the metrics assessment system, i.e. 20; the output layer is the overall performance of a whole, i.e. the number of nodes in one output layer.

(3) Determining the number of neurons in the implicit layer

**Figure 2:**
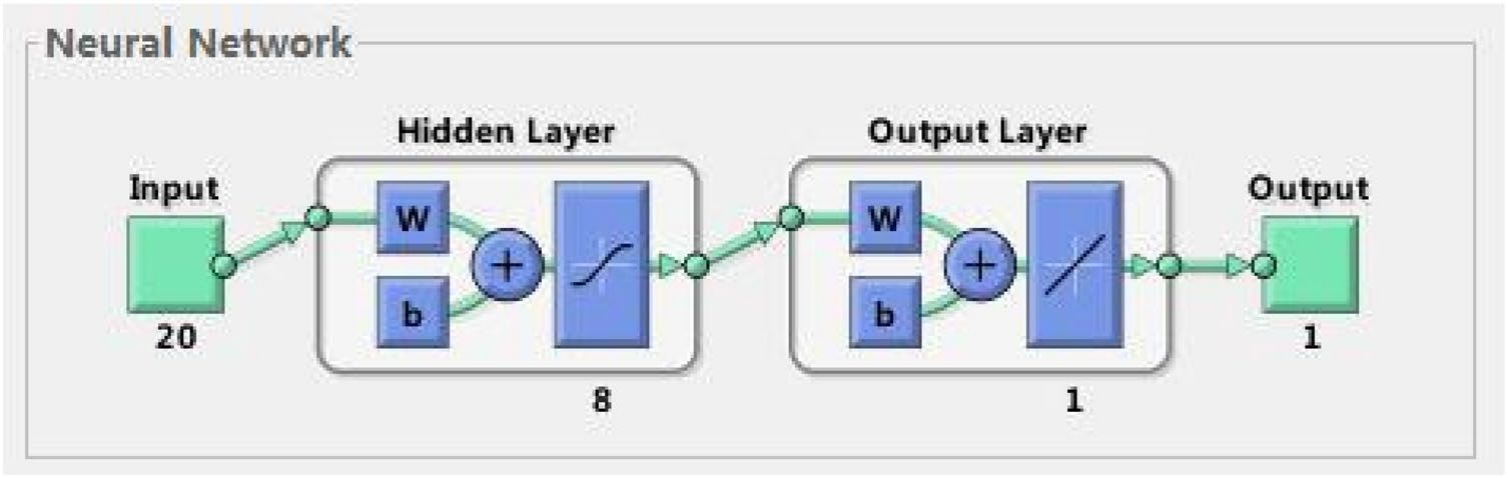
Network structure of the integrated firm performance evaluation model.

The design of nodes in the hidden layer is complex and there is no uniform theoretical basis on which one can draw. Since too small a number of nodes in the implicit layer can cause instability in the model; too much of it tends to produce overlearning. First, we cited several empirical formulas for calculating the number of nodes in the hidden layer and substituted them to obtain an approximate range of the number of nodes in the hidden layer, as K = n*l + a, where k is the number of nodes in the hidden layer, n is the number of nodes in the input layer, l is the number of nodes in the output layer,and a is a constant between 1 and 10. In general, in a three-level deep neural network, the number of nodes in the hidden layer is n-1. Secondly, a trial-and-error method was used to test the number of hidden layer nodes in the network, starting from the node with the lowest number of nodes and increasing the number of training samples in turn. The performance of the network was evaluated according to the learning time, number of iterations and the size of network errors, and eight hidden layer nodes were obtained. The network architecture is shown in Fig. 2.

As shown in Fig. 2, the enterprise performance evaluation model should achieve the following objectives, standards, and methods of public evaluation; Public evaluation process and results; Adhering to this principle can eliminate the doubts of the evaluation object about the performance evaluation work, improve the credibility of the performance evaluation results: it is beneficial for the evaluation object to see their own problems and gaps, then find the goal and direction of effort, and stimulate the enthusiasm for further improvement of work; At the same time, it can also enhance the sense of responsibility of the human resources department, prompting them to continuously improve their work and improve the quality of work.

(4) Determining the parameters of the transfer function

On this basis, a transfer function based on the implicit layer is constructed. Among the Sigmoid functions, the most commonly used is the tansig function, while the output layer is purelin, which is a purely linear function. In practical applications, the Sigmoid and linear functions are generally chosen.

Performing certain predetermined transformations on the input data of a neural network is called preprocessing, which is very practical in neural networks (deep learning) and its effectiveness has been proven in numerous experiments such as improving recognition performance and learning efficiency. In fact, many preprocessors consider the overall distribution of the data. For example, using the overall mean or standard deviation of the data to move it around and distribute it around 0, or normalizing it to control its extension within a certain range. In addition, there is also a method of homogenizing the overall distribution shape of the data, namely data whitening. The network is designed with a training count of 1000 and the learning rule uses the traingdx function, which combines dynamic and adaptive learning with a rate gradient reduction method that can increase the training speed and improve the stability of the system while minimising the local miniaturisation of the results.

### Ambidextrous innovation and social network impact data processing

**Figure 3:**
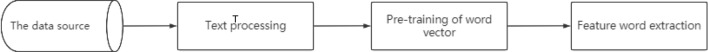
Data processing logic diagram.

There are currently no publicly available datasets on the Internet that can be used for corporate project performance analysis, so a web crawler is used to obtain them from a corporate information platform. This paper uses a Python crawler to crawl from this information platform. The data obtained includes the company's performance data, information on the company's ambidextrous innovation characteristics, and information on the company's social network characteristics. The data processing logic diagram for the ambidextrous innovation and social network features is shown in Fig. 3.

## Implementation and analysis of results

### Experimental environment and basic parameter settings

In this experiment, we use a Python + tensorflow program developed by Google, which provides an interface between C +  + and Python and supports mainly Linux and Mac operating systems. In a graph, a node represents a numerical operation, or the input and output of a piece of data. Edges represent relationships between nodes, and multiple arrays of bits (tensors, tensors) are transferred between operations, and tensors flow through the graph, which is how TensorFlow got its name. Nodes are assigned to computational devices in different (inter-node), parallel (in-node) ways as the data flow is passed from one side of the connected node, and the architecture allows us to utilise the same API for operations on individual CPUs, GPUs, servers and mobile devices. This paper uses a Linux development environment, and in Table 1 shows the specific lab environment.

**Table 1:** Experimental environment.

CPU	i5-6500 CPU @ 3.20 GHz
GPU	GTX750 ti@2 GB
Memory	8 GB
Operating systems	Linux mint 18
Deep learning frameworks	GoogleTensorFlow (r0.12)
Programming Languages	Python 2.7

### Operation of the deep neural network model

**Figure 4:**
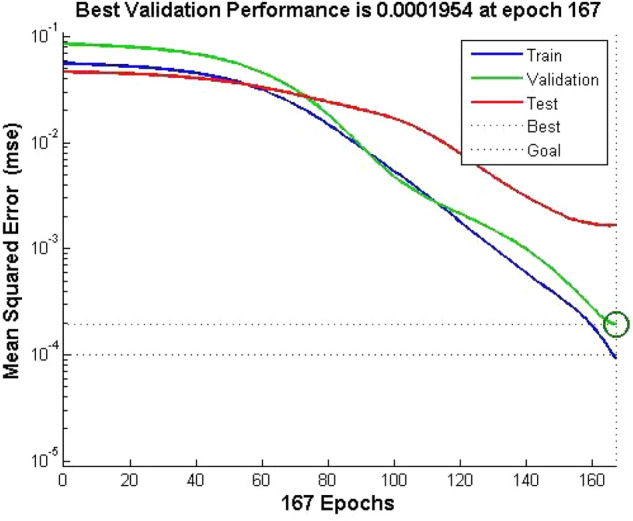
Deep neural network training results.

With the changes in the market environment and the intensification of competition, enterprises need to find new ways to improve their performance. Traditional experience and intuition cannot meet the needs of modern enterprise development, so more refined methods are needed to optimize various links, improve enterprise efficiency, and enable them to gain advantages in fierce market competition. The network model was trained and tested through the selection of learning samples for the model. With 167 steps of the network run, the squared and MSE of the model error were smaller than the target error, and good simulation results were obtained and better results were obtained. The training effect of its deep neural network is shown in Fig. 4.

The trained network produces a stable threshold of weights after training, and then the corresponding result, the overall performance score of the business, is obtained after inputting new data.

**Figure 5:**
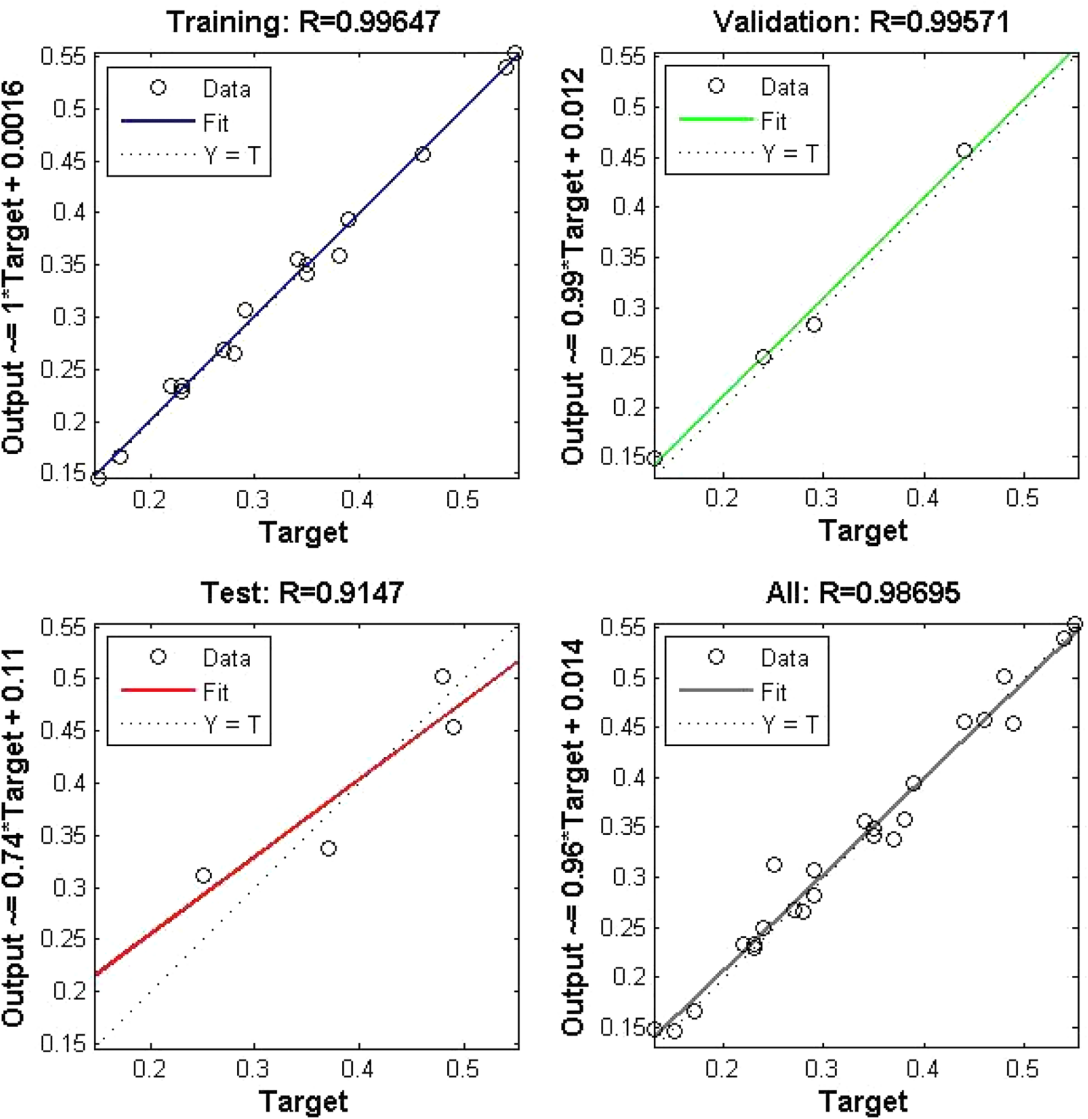
Linear regression analysis of the actual and desired outputs of the deep neural network.

A call is made to the saved model and the data from the sample is fed into the model to obtain the results of the experiment. The results of the test are shown in Fig. 2 and the errors are shown in Fig. 5. A comparison of the expected and actual results shows that the deviation is within an acceptable range.

The fact that it is within the acceptable range, realistic and logical, indicates that the model has a good generalisation function and can be widely used in the overall performance assessment of companies (Table 2).

**Table 2:** Comparison of the expected output of the deep neural network with the actual output.

Sample predictions	A	B	C	D	E
Expected output	0.4292	0.3296	0.2069	0.2700	0.1323
Actual output	0.4347	0.3287	0.2074	0.2426	0.1511
Absolute error	0.0055	0.0009	0.0005	0.0274	0.0188
Relative error	1.28%	0.28%	0.23%	10.13%	14.21%
Mean absolute error			0.0106		
Mean relative error			5.23%		

The results show that the absolute deviation between the results obtained with the deep neural network model and the expected results is about 0.01, the maximum relative error is 14.21%, the minimum is 0.23% and the average relative error is 5.23%. It can be seen that this neural network is efficient in evaluation and has a low error, which can solve this problem to a certain extent.

It will be further promoted in the comprehensive performance evaluation of companies. In the future, when conducting comprehensive performance evaluation of other companies, it is only necessary to invoke this network model and input the data of each of the above indicators to obtain their comprehensive performance scores quickly through the network model.

### Analysis of the results of the impact of ambidextrous innovation on business performance

This time, the main analysis is the comprehensive performance, using the crawler data as the support of the data source, and then its model training and running processing of the data, after running the data fed back from it using SPSS24.0 statistical software to conduct a descriptive statistical analysis of ambidextrous innovation and firm performance, so as to derive the mean and standard deviation of each dimension of ambidextrous innovation and firm performance, to understand the enterprise's ambidextrous innovation and firm performance. The descriptive statistics table is shown in Table 3.

**Table 3:** Descriptive statistics.

Indicators	Average value	Standard deviation
Ambidextrous innovation	3.5796	0.8489
Task performance	3.3320	1.0352
Relationship performance	3.2289	0.9894
Overall performance	3.2805	0.9492

Table 3 shows that the mean value of ambidextrous innovation is 3.58, which is an average level of ambidextrous innovation. The mean values for the two dimensions of firm performance are 3.33 and 3.22 respectively, while the mean value for overall firm performance is 3.28, which is also average. In terms of standard deviation, the standard deviation of ambidextrous innovation and overall performance is low, suggesting that the sample firms are relatively consistent in their views on ambidextrous innovation.

**Figure 6:**
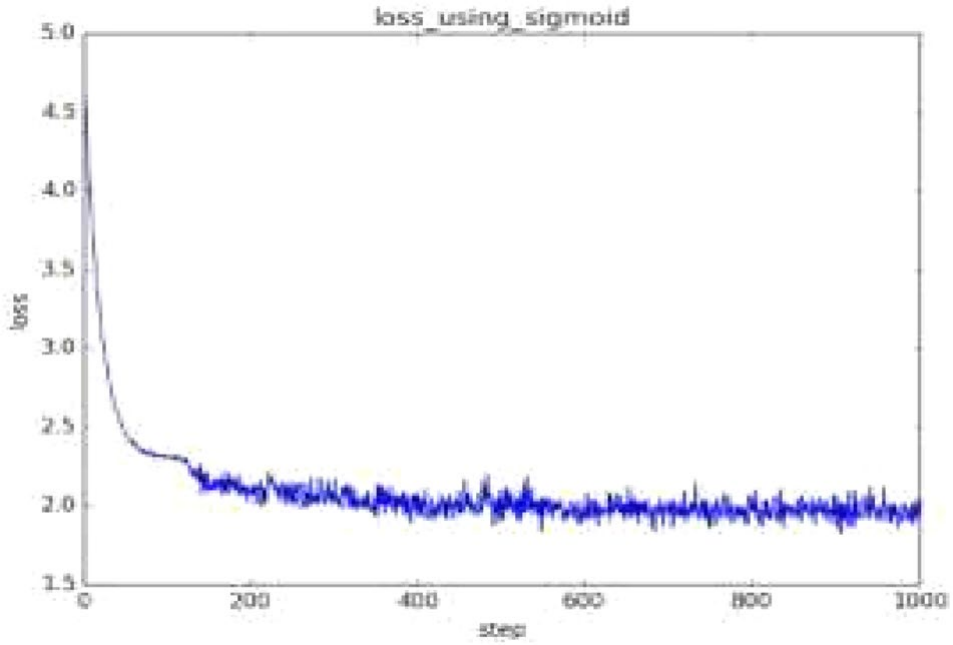
Visualisation of binary innovation.

On this basis, the different dimensions of binary innovation in the sample data were modified by upgrading the data, and then the deep neural network model was used to process the data, and then the data was visualised using echarts. The reason for this is that as the proportion of ambidextrous innovation increases, the proportion of other components decreases accordingly, so the overall firm performance also decreases. The visualisation is shown in Fig. 6.

### Analysis of the results of the impact of social networks on business performance

This time, the main analysis is the comprehensive performance, using the crawler data as the support of the data source, and then its model training and running processing of the data, after running the data fed back from it using SPSS24.0 statistical software to conduct a descriptive statistical analysis of the enterprise social network and firm performance, so as to derive the mean and standard deviation of each dimension of the enterprise social network and firm performance, to understand the enterprise The descriptive statistics table is shown in Table 4. The descriptive statistics table is shown in Table 4.

**Table 4:** Descriptive statistics.

Indicators	Average value	Standard deviation
Network size	3.4524	1.2714
Network centrality	3.4829	1.2412
Network Heterogeneity	3.5051	1.2311
Task performance	3.3120	1.0352
Relationship performance	3.3489	0.9894
Overall performance	3.3305	0.9492

Table 4 shows that the lowest mean value for the social network dimensions was 3.45 and the highest mean value was 3.51. The mean value for the overall social network was 3.48, which is an average level of social network, with the lowest score for network size. The mean values for the two dimensions of corporate performance were 3.31 and 3.34 respectively, and the mean value for overall job performance was 3.33, which was also average, with less significant differences between the dimensions. In terms of standard deviation, the low standard deviation for social networks and job performance suggests that the sample companies are relatively consistent in their views on social networks.

**Figure 7:**
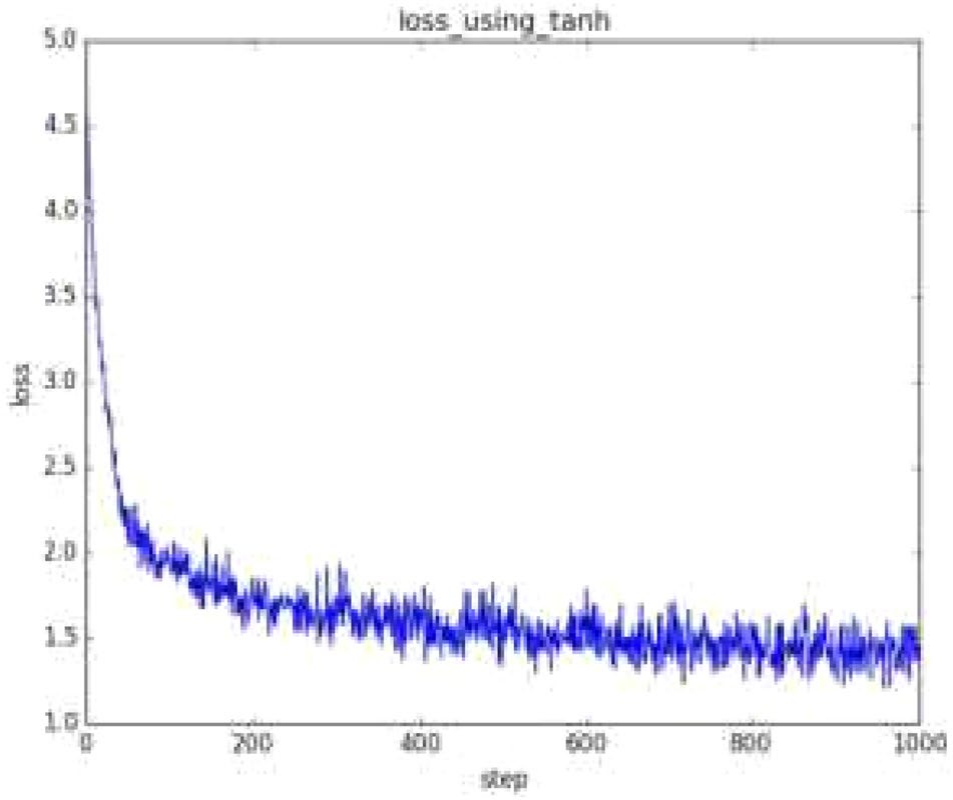
Social network visualisation.

On this basis, the different dimensions of the social network of the sample data were modified by upgrading the data, and then the deep neural network model was used to process the data, and then echarts was used to visualise the data. It can be clearly seen from Fig. 7 that the initial mean value of firm performance increased a lot by upgrading the different dimensions of the social network again, but as the proportion of the social network increased, the enterprise The reason for this is that as the proportion of social networks increases, the proportion of other components decreases accordingly, so the overall firm performance also decreases gradually. The visualisation is shown in Fig. 7.

Based on the influence of deep neural network, dual innovation and social network on enterprise performance, this is a complex problem involving multiple fields, which needs to be studied in combination with deep learning, innovation management, social network and other aspects.

Firstly, in terms of deep learning, a deep neural network can be constructed to mine enterprise data, extract useful information from it, and improve the company's insight and predictive ability towards market trends and customer needs. Through deep learning methods, factors such as consumer behavior and the operational status of collaborative innovation mechanisms can be more accurately analyzed and identified, providing enterprises with more accurate strategic decisions.

Secondly, in terms of dual innovation, the stakeholders of the enterprise, including employees, investors, consumers, suppliers, etc., have the potential for innovation. They can convey their innovative opinions to the enterprise and promote the rapid recognition of related products and services in the market. At this point, the internal innovation environment of the enterprise is not only dominated by internal employees, but also achieves overall innovation through overseas collaboration, cooperation and sharing. Therefore, the inspiration of dual innovation can drive enterprise development through multiple channels such as consumer feedback, employee knowledge sharing, and investor guidance.

Finally, in terms of social networks, there have been many studies proving that social media platforms play a very important role in the marketing and brand building of enterprise business. Interact and communicate with existing customers or potential users through the internet.

## Conclusion

Internal factors such as organizational structure and leadership style also have a significant impact on corporate performance. Specifically, a flexible organizational structure and open leadership style can create more opportunities and freedom for enterprises, effectively driving innovation and improving performance. The above are some of the main findings on the impact of dual innovation and social networks based on deep neural networks on corporate performance, which will provide important reference value for enterprise management and decision-making. Corporate performance assessment is a major approach and tool for business management and has now received widespread attention from both the corporate and theoretical communities. For companies to gain a strong competitive advantage in an integrated global economy, they must make a proper assessment of their performance. In this paper, we have analysed the company's performance through in-depth neural networks, and found that ambidextrous innovation and social networks have a great contribution to the company's performance, but if the improvement is too large, it will lead to the reduction of other factors and thus the overall corporate performance, so ambidextrous innovation and social networks are able to help the company achieve a greater advantage in the market within a certain interval.

### Informed Consent

This study does not involve human participants (including the use of tissue samples).

## Data Availability

The datasets used and/or analysed during the current study available from the corresponding author on reasonable request.
